# Cleanliness in Public Buildings

**Published:** 1917-11-24

**Authors:** F. Fowell

**Affiliations:** Secretary. Broadmead House, Panton Street, Haymarket


### Cleanliness in Public Buildings.

*To the Editor of* The Hospital.

Sir,—I am very glad to see that in your issue of October 20 you return to the question of the ventilation of the picture-house under the heading "The Report of the Cinema Commission," because I am convinced that concentration of attention on these problems can only be of benefit both to the trade and the public.

But your little reference to my last communication, which I thought you had already answered, conveys an unwitting misapprehension. I never attempted to plead anything in extenuation of the fact that vacuum cleaners are not "usually in use in picture theatres," for the simple reason that they are almost invariably used. There are small out-of-the-way picture theatres, just as there are small out-of-the-way hospitals, where the most modern requirements are not always ideally complied with. My letter was merely an attempt to show that this question of cleanliness in public buildings (not merely in cinema theatres) is not quite so simple as some of my, and probably your, correspondents seem to think. I have never claimed that the older theatres are as perfect as the newer ones, and I am sure you would not be stupid enough to suggest that commercial buildings can be scrapped and rebuilt from year to year to keep pace with the advances in hygiene. All that can be asked is that as new buildings are erected they shall be as perfect as possible according to contemporary ideas, and a moderate progressive scale should be adopted in regard to existing buildings.—Yours faithfully,

Cinematograph Trade Council.
F. Fowell, Secretary.

Broadmead House, Panton Street, Haymarket,
November 14, 1917.

